# Identifying diagnostic DNA methylation profiles for facioscapulohumeral muscular dystrophy in blood and saliva using bisulfite sequencing

**DOI:** 10.1186/1868-7083-6-23

**Published:** 2014-10-29

**Authors:** Takako I Jones, Chi Yan, Peter C Sapp, Diane McKenna-Yasek, Peter B Kang, Colin Quinn, Johnny S Salameh, Oliver D King, Peter L Jones

**Affiliations:** The Wellstone Program & The Department of Cell and Developmental Biology, University of Massachusetts Medical School, 55 Lake Avenue North, Worcester, MA 01655 USA; Key Lab of Swine Genetics and Breeding, Ministry of Agriculture, College of Animal Science and Technology, Huazhong Agricultural University, Wuhan, 430070 P.R. China; The Department of Neurology, University of Massachusetts Medical School, 55 Lake Avenue North, Worcester, MA 01655 USA; Department of Pediatrics, Division of Pediatric Neurology, University of Florida College of Medicine, 1600 SW Archer Road, Gainesville, FL 32607 USA; Department of Neurology, Hospital of the University of Pennsylvania, 3400 Spruce St, 3 Gates, Philadelphia, PA 19104 USA; The Eunice Kennedy Shriver National Institute of Child Health and Human Development Sen. Paul D. Wellstone Muscular Dystrophy Cooperative Research Center, University of Massachusetts Medical School, 55 Lake Avenue North, Worcester, MA 01655 USA

**Keywords:** Bisulfite sequencing, DNA methylation, D4Z4, Disease diagnostics, DUX4, Epigenetics, Facioscapulohumeral muscular dystrophy, FSHD1, FSHD2

## Abstract

**Background:**

Facioscapulohumeral muscular dystrophy (FSHD) is linked to chromatin relaxation due to epigenetic changes at the 4q35 D4Z4 macrosatellite array. Molecular diagnostic criteria for FSHD are complex and involve analysis of high molecular weight (HMW) genomic DNA isolated from lymphocytes, followed by multiple restriction digestions, pulse-field gel electrophoresis (PFGE), and Southern blotting. A subject is genetically diagnosed as FSHD1 if one of the 4q alleles shows a contraction in the D4Z4 array to below 11 repeats, while maintaining at least 1 repeat, and the contraction is in *cis* with a disease-permissive A-type subtelomere. FSHD2 is contraction-independent and cannot be diagnosed or excluded by this common genetic diagnostic procedure. However, FSHD1 and FSHD2 are linked by epigenetic deregulation, assayed as DNA hypomethylation, of the D4Z4 array on FSHD-permissive alleles. We have developed a PCR-based assay that identifies the epigenetic signature for both types of FSHD, distinguishing FSHD1 from FSHD2, and can be performed on genomic DNA isolated from blood, saliva, or cultured cells.

**Results:**

Samples were obtained from healthy controls or patients clinically diagnosed with FSHD, and include both FSHD1 and FSHD2. The genomic DNAs were subjected to bisulfite sequencing analysis for the distal 4q D4Z4 repeat with an A-type subtelomere and the DUX4 5’ promoter region. We compared genomic DNA isolated from saliva and blood from the same individuals and found similar epigenetic signatures. DNA hypomethylation was restricted to the contracted 4qA chromosome in FSHD1 patients while healthy control subjects were hypermethylated. Candidates for FSHD2 showed extreme DNA hypomethylation on the 4qA DUX4 gene body as well as all analyzed DUX4 5’ sequences. Importantly, our assay does not amplify the D4Z4 arrays with non-permissive B-type subtelomeres and accurately excludes the arrays with non-permissive A-type subtelomeres.

**Conclusions:**

We have developed an assay to identify changes in DNA methylation on the pathogenic distal 4q D4Z4 repeat. We show that the DNA methylation profile of saliva reflects FSHD status. This assay can distinguish FSHD from healthy controls, differentiate FSHD1 from FSHD2, does not require HMW genomic DNA or PFGE, and can be performed on either cultured cells, tissue, blood, or saliva samples.

**Electronic supplementary material:**

The online version of this article (doi:10.1186/1868-7083-6-23) contains supplementary material, which is available to authorized users.

## Background

**Figure 1:**
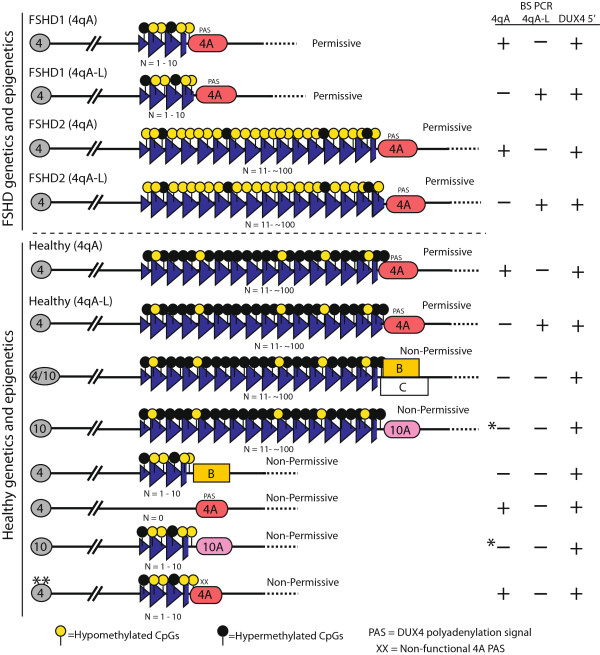
**The molecular signatures of FSHD are complex, as illustrated by healthy and FSHD-type chromosomes.** In the general healthy population, each chromosome 4q arm has a large polymorphic array of D4Z4 repeats containing more than 10 RUs. In FSHD1, there is a dominant contraction of one 4q array to between 1 and 10 D4Z4 repeat units, whereas FSHD2 is contraction-independent. There are two main allelic variants in the subtelomere distal to the array, termed A and B. A rare third classification of subtelomere, termed C, is used for subtelomeres that do not hybridize with probes for A or B due to distal sequence changes [18]. In some instances, the distal-most repeat fragment of the 4q D4Z4 array contains additional ~2 kb of D4Z4 sequence, resulting in a longer terminal RU in *cis* with a 4qA subtelomere; this type of 4qA allele is referred to as 4qA-L [15]. Both FSHD1 and FSHD2 are exclusively linked to the 4qA subtelomere allelic variants containing a PAS for the *DUX4-fl* mRNA [12, 15]. In addition, both FSHD1 and FSHD2 require the epigenetic disruption of the D4Z4 array to a less methylated and more relaxed chromatin state. Results of the described bisulfite sequencing assays are indicated by “+” if a bisulfite (BS) PCR product is produced and “–” if no BS PCR product is produced. *On rare occasions, due to primer degradation, a 10qA BS PCR product is detected; however, sequencing eliminates these from analysis. **Diagnosis of this healthy chromosome requires genomic PCR and sequencing of the 4qA subtelomere to identify a non-permissive 4qA PAS.

Facioscapulohumeral muscular dystrophy (FSHD) is the most prevalent myopathy that indiscriminately affects males and females of all ages [1–3]. Although clinical muscle weakness typically manifests in the second or third decade of life, there is great variability in clinical severity, from a severe infantile form to individuals who remain asymptomatic throughout their lives [1, 2, 4–7]. Genetically, there are two classes of FSHD that are both linked to the chromosome 4q35 D4Z4 macrosatellite array (Figure 1). In the healthy population, these polymorphic regions exist as 11 or more repeat units (RUs) on each chromosome (24 to 35 RUs on average and up to ~120 [8]). A patient is genetically diagnosed with FSHD1 if pulse-field gel electrophoresis (PFGE) analysis indicates that one of the 4q alleles has a contraction in the D4Z4 array to below 11 RUs, while maintaining at least 1 RU, and the contraction is in *cis* with a FSHD-permissive 4A-type subtelomere containing a functional polyadenylation signal (PAS) for the pathogenic *DUX4-fl* (*DUX4*-full length) mRNA [9–15]. In contrast, the far less common form, FSHD2, is highly similar to FSHD1 in clinical presentation, yet it is contraction-independent and cannot be diagnosed or excluded by this common molecular diagnostic procedure [16, 17]. However, as with FSHD1, FSHD2 also requires a disease-permissive 4A-type subtelomere allele distal to the D4Z4 array on at least one 4q chromosome [15], suggesting the expression of *DUX4-fl* is likely a key mechanism in both forms of FSHD. Interestingly, the majority of 4A-type subtelomeres are, in fact, disease-permissive [15, 18]. FSHD1 and FSHD2 are also linked by epigenetic deregulation, typically assayed by DNA methylation analysis, of the 4qA FSHD-permissive allele [17, 19]. In healthy subjects, both copies of the 4q35 D4Z4 array as well as both copies of the 10q26 D4Z4 array have hypermethylated DNA (>35% CpGs assayed are methylated). In FSHD1 patients, the contracted 4q35 D4Z4 array exhibits DNA hypomethylation while the non-contracted 4q35 allele remains hypermethylated [17, 19, 20]. FSHD2 patients do not have contractions in either 4q35 array; however, both 4q35 D4Z4 arrays and both 10q26 D4Z4 arrays are severely hypomethylated (<25% CpGs assayed are methylated) due to mutations in the *SMCHD1* (structural maintenance of chromosomes flexible hinge domain containing1) gene, or other yet-to-be-identified epigenetic modifiers of D4Z4 repression [17, 19, 21]. These DNA hypomethylation signatures are specific to FSHD, as DNA methylation patterns of the 4q/10q D4Z4 arrays in other muscular dystrophies are similar to those found in healthy subjects [17].

The typical genetic diagnosis for FSHD1 is complex [12, 22]. It first requires careful isolation of 40 to 50 μg of very high molecular weight (HMW) DNA from peripheral blood mononuclear cells (PBMCs) obtained from fresh blood samples [18]. The purified genomic DNA is then embedded in agarose for in-gel digestion with combinations of several restriction enzymes. The agarose-DNA plugs are subjected to PFGE, Southern blotting, and hybridizations with DNA probes: the p13E-11 probe to identify the size of each 4q35 and 10q26 array [23], and probes for the generally permissive A-type subtelomere and the non-permissive B-type subtelomere to identify the haplotype of 4q35 and 10q26 chromosomes, respectively. Recently, an alternative fluorescent cell-based technique, termed molecular combing, was developed to identify an FSHD1 deletion on a 4qA chromosome [24]. The additional information one obtains from these assays includes interchromosomal rearrangements and potential somatic mosaicism; however, these assays are incapable of identifying a functional *DUX4-fl* PAS [12, 22, 25, 26]. The ~5% of clinical FSHD patients that do not have an FSHD1-sized pathogenic 4qA allele are candidates for FSHD2, but neither assay can identify these individuals as FSHD2 as opposed to another myopathy with similar clinical symptoms. Sequencing the *SMCHD1* gene for known FSHD2 mutations in candidates with permissive 4A-type subtelomeres will identify many, but not all, FSHD2 subjects [21, 27, 28].

**Figure 2:**
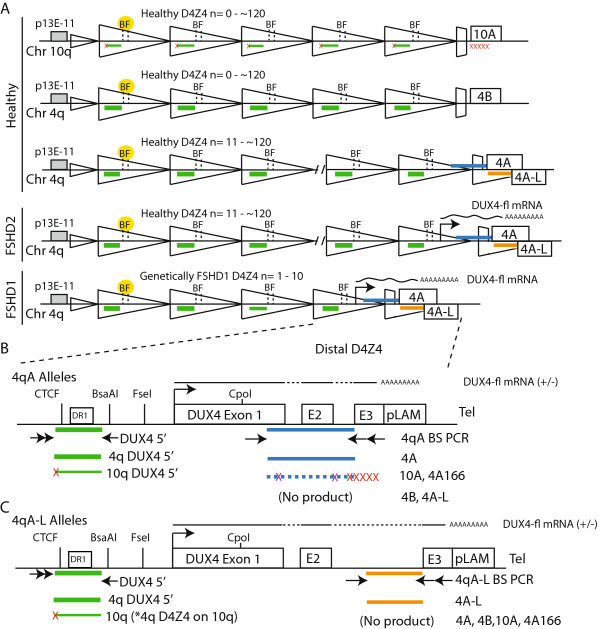
**Schematic for bisulfite sequencing (BSS) analysis of FSHD-associated 4qA chromosomes and 4q D4Z4 repeat units. (A)** Cartoon depicting the location of bisulfite (BS) PCR products for the 4qA BSS assay (blue), the 4qA-L BSS assay (orange), and the DUX4 5’ BSS assay (green). For the DUX4 5’ reaction, the nested primer has a preference for a 4q D4Z4 polymorphism (red “x”); however, a fraction of D4Z4 units are amplified from chromosome 10q arrays (denoted by thin green lines), (*) including chromosome 4q-type D4Z4 units present on chromosome 10q due to *trans* chromosomal rearrangements found in ~6% of subjects [18]. The proximal *Bsa*AI and *Fse*I methylation-sensitive restriction enzyme sites analyzed by Southern blotting are indicated (B and F, respectively) and highlighted in yellow. **(B and C)** Diagrams of the distal-most D4Z4 repeat that produces the polyadenylated *DUX4-fl* mRNA and is analyzed in the **(B)** 4qA BSS assay and **(C)** 4qA-L BSS assay. Arrows indicate BS PCR primers and red “X” indicates sequence differences with 4qA; rare 10A or 4A166 products amplified in the absence of 4A alleles and due to primer degradation are detected and eliminated from analysis by specific sequence polymorphisms (Additional file 1: Figure S1). Neither 4qA nor 4qA-L BSS assay amplifies the 4qB allelic variant.

Here, we designed a new analytical method to address several issues critical to FSHD clinicians and researchers. We first sought to develop a molecular assay that could readily distinguish FSHD2 from FSHD1 and other limb-girdle-like myopathies. Ideally, the assay would not require isolation of HMW DNA and could be performed from saliva samples for sampling convenience. Since the majority of 4A subtelomeres are disease permissive in all forms of FSHD [15, 18], and distinct 4q and 10q D4Z4 hypomethylation profiles are characteristic for FSHD1 and FSHD2, we have developed an assay using a set of three PCR-based bisulfite sequencing (BSS) reactions that together identify the epigenetic signatures for FSHD1, FSHD2, and unaffected subjects. Our D4Z4 BSS analysis can clearly distinguish each form of FSHD from the others by assessing the overall DNA methylation status of both 4q35 D4Z4 arrays, providing a precise DNA methylation pattern for the distal-most D4Z4 repeat on the contracted allele, and having specificity for the generally permissive 4A subtelomere (Figure 2). Furthermore, this analysis does not require HMW DNA or any special equipment and can be performed on as little as 1 μg of genomic DNA isolated from blood, tissue, or saliva, using standard molecular biology techniques. Importantly, we show that the DNA methylation profiles of the 4q35 D4Z4 in saliva yield comparable results to those in blood. Although we used traditional subcloning and Sanger sequencing for proof-of-principle, our method can easily be modified for high-throughput acquisition and analysis using bar-coded oligonucleotides and next-generation sequencing methods. This method will identify characteristic FSHD epigenetic signatures in *cis* with a 4A subtelomere, distinguish FSHD2 from FSHD1 subjects, and enable epigenetic studies on the FSHD pathogenic locus. In addition, although it does not provide the size of a D4Z4 contraction or identify functional PAS, this method, when used as a diagnostic tool with patients that exhibit a clinical manifestation of neuromuscular disease, will likely out-perform current PFGE or molecular combing diagnostics by not only accurately characterizing patients with short D4Z4 arrays on permissive 4qA chromosomes as having FSHD1, but also accurately preventing many patients with contracted arrays on non-permissive chromosomes from being misdiagnosed as FSHD1.

## Results and discussion

### Development of a combined distal 4qA-specific and 4q/10q 5’ D4Z4 DNA methylation assay

**Figure 3:**
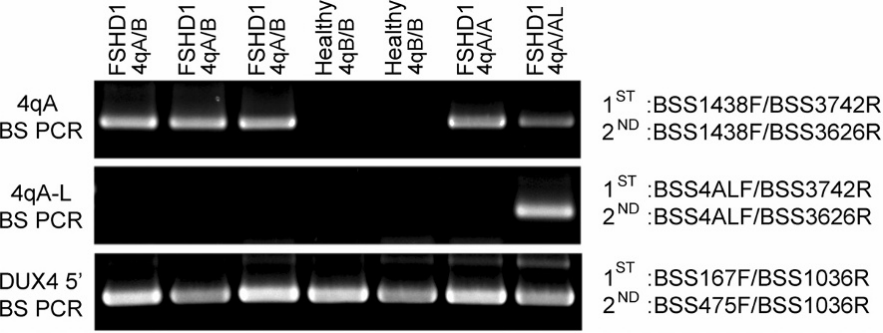
**Bisulfite (BS) PCRs using genomic DNAs from subjects with a range of 4q allelic combinations show the specificity of the three bisulfite sequencing (BSS) assays.** Nested PCRs were performed using BS-converted genomic DNAs from seven subjects, five FSHD1 and two healthy, with varying 4q haplotypes (4qA/A, 4qA/B, 4qB/B, and 4qA/A-L, as indicated). The primer sets used are indicated to the right of each panel. The 4qA BS PCR (upper panel) amplified a product from all five subjects possessing at least 1 4qA allele and did not amplify any detectable product from the two subjects lacking a 4qA allele. The 4qA-L BS PCR (middle panel) only amplified a product from the one subject possessing a 4qA-L allele. The DUX4 5’ BS PCR (lower panel) amplified a product from all seven subjects. The identities of all BS PCR products were confirmed by sequencing.

Dramatic epigenetic differences at the 4q35 D4Z4 repeat array between healthy and disease states distinguish FSHD1 and FSHD2 from unaffected individuals. Epigenetic differences at the non-contracted 4q35 D4Z4 and the 10q26 D4Z4 arrays distinguish FSHD2 from FSHD1 and other myopathies. In all forms of FSHD, it is the distal 4q35 D4Z4 in *cis* with a disease-permissive 4A subtelomere that produces the pathogenic *DUX4-fl* mRNA [15]. However, this pathogenic D4Z4 repeat has never been specifically analyzed in FSHD1 or FSHD2 [17, 19, 20, 29]. Therefore, in order to study epigenetic changes at the disease-relevant D4Z4 repeat, we developed two BSS assays that specifically analyze the distal 4qA- or 4qA-L-associated D4Z4 RU (Figure 2). Utilizing polymorphisms in the BSS PCR primers that are exclusive to the disease-permissive 4A subtelomere and not found in 10A, the 4qA BSS assay analyzes the DNA methylation status of 56 CpGs (Additional file 1: Figure S1A) in the distal D4Z4 RU in *cis* with a 4A subtelomere (Figure 2B). The 4qA bisulfite (BS)-PCR product is amplified from all BS-converted genomic DNAs from subjects possessing at least one 4qA allele (Figure 3, upper panel). The D4Z4-4A fragments were sequenced and, importantly, all 56 CpGs predicted by the reference sequence were accounted for in >90% of the analyzed sequences from these clones, confirming the specificity of the reaction for the distal 4qA-derived D4Z4. The 4qA-L BSS assay utilizes the same 4A subtelomere-specific reverse BS PCR primers as above; however, these are paired with a 4qA-L-specific forward BS PCR primer. The 4qA-L BSS assay analyzes the DNA methylation status of 30 CpGs (Additional file 1: Figure S1D) in the distal D4Z4 repeat on 4qA-L chromosomes (Figure 2C). The 4qA-L BS PCR product was amplified exclusively from BS-converted genomic DNAs from the one subject possessing a 4qA-L allele and not from any of the six subjects lacking a 4qA-L allele (Figure 3, middle panel). The 4qA-L fragment was sequenced and all 30 CpGs predicted by the reference sequence were accounted for in 100% of the analyzed sequences from these clones, confirming the specificity of the reaction for the distal 4qA-L-derived D4Z4. Neither the 4qA BS PCR nor the 4qA-L BS PCR produced a product from genomic DNAs isolated from either of the two healthy subjects with 4qB/B haplotypes. It is worth noting that due to the 4qA-specific SNPs residing at the 3’ end of the 4qA BS PCR oligonucleotide primers, multiple rounds of primer freeze-thaw, which leads to partial primer degradation, results in a loss of specificity and a consequent amplification of minor products from genomic DNAs lacking the 4qA allele (Additional file 1: Figure S1B). Sequence analysis of these rare amplicons from 4qB/B samples identified them as either a 10qA product (Additional file 1: Figure S1C) or a non-specific product not derived from any D4Z4 or 4qA/B allelic variant. In addition, since the BSS analysis is sequence-based and not product-based (as in qPCR or Southern blotting), any rare non-specific or 10qA amplifications present are easily identified and removed from the analysis. We conclude that these assay conditions amplify the distal D4Z4 sequence from 4qA chromosomes or 4qA-L chromosomes, depending on the assay, and neither assay amplifies 10A or 4B subtelomere-containing chromosomes.

To complement the distal D4Z4 methylation analysis and provide the context for both 4q35 D4Z4 arrays that is important for the determination of FSHD2 status, we designed a third BSS analysis upstream of the DUX4 open reading frame (referred to as the DUX4 5’ BSS assay). This assay analyzes the methylation status of 59 CpGs preferentially in 4q35 D4Z4 RUs but also in 10q35 D4Z4 RUs (Figures 2 and Additional file 1: Figure S1E). This DUX4 5’ region can be amplified from all 4q35 and 10q26 D4Z4 RUs, does not amplify homologous D4Z4s elsewhere in the genome [30], and encompasses a putative CTCF binding site and the DR1 region found to be hypomethylated in all 4q and 10q D4Z4 RUs in FSHD2 cells [29, 31]. As anticipated, all seven of the BS-converted genomic DNAs were successfully amplified using this protocol (Figure 3, lower panel), validating the integrity of the BS-converted DNAs from the two healthy subjects. Analysis of the DUX4 5’ BSS products revealed that all 59 of the CpGs predicted by the reference sequence were accounted for in all sequences in this assay, confirming that these sequences were derived from 4q/10q D4Z4 RUs, which characteristically have very few polymorphisms, and not from homologous D4Z4s located elsewhere in the genome that contain numerous sequence polymorphisms [30]. Thus, combining the DUX4 5’ BSS and 4qA/4qA-L BSS assays provides a detailed analysis of the DNA methylation status of the pathogenic distal 4qA D4Z4 RU in the context of overall 4q/10q D4Z4 DNA methylation.

### Characterization of healthy and FSHD1 DNA methylation patterns in the distal D4Z4 repeat unit using blood and saliva

Epigenetic marks often show tissue specificity; thus, it is very important to carefully examine and compare each locus of interest when performing epigenetic studies on genomic DNAs isolated from different tissue sources [32]. Since FSHD is a myopathy and the pathogenic *DUX4* mRNA is expressed predominantly in skeletal muscle [1, 33], the epigenetic status of myocytes is of particular interest. However, muscle biopsies require participants to visit a hospital or clinic, and can be expensive, painful, and difficult to obtain from FSHD patients of any age already exhibiting muscle atrophy. Fortunately, in FSHD1 and FSHD2, the DNA methylation status of the 4q35 D4Z4 is similar between PBMCs and myogenic cells [17]. For example, in FSHD1, the proximal repeats of the D4Z4 array on the contracted 4q35 allele are significantly hypomethylated in both PBMCs and myogenic cells compared to the non-contracted allele or healthy controls [17]. In order to assess the DNA methylation status of the pathogenic distal 4q35 D4Z4 repeat, we used our 4qA and 4qA-L BSS assays to analyze the distal D4Z4 in PBMCs from FSHD1 patients and healthy first-degree relatives. In addition, we are interested in analyzing the epigenetic signatures of large numbers of family members over time, including healthy individuals, some of whom may be identified as potential asymptomatic carriers. Therefore, in addition to testing our assay on genomic DNA isolated from PBMCs, we performed our analysis on saliva samples obtained from the same subjects for a comparison. The advantage of saliva samples is that they can be collected without additional help, there is no needle injection, and collection kits can be mailed to subjects who have undergone informed consent, with the stable 2 mL sample returned by standard mail. This type of testing would be particularly useful for children and in communities or countries where access to a phlebotomist is limiting or relatively expensive and/or standard genetic testing by PFGE or molecular combing is cost-prohibitive or unavailable.

**Figure 4:**
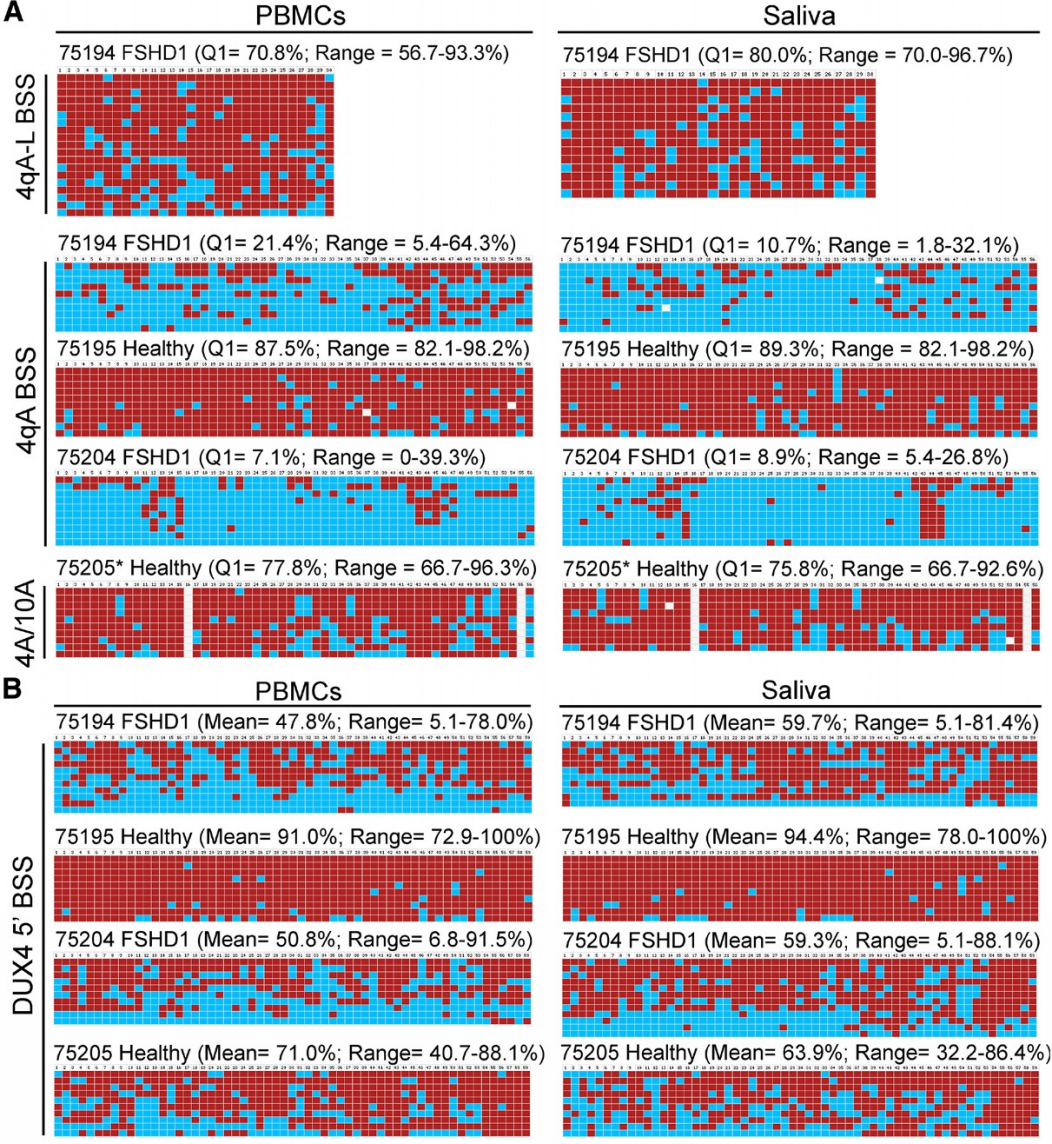
**BSS analysis identifies distinct epigenetic signatures for FSHD1 and healthy controls that are similar between genomic DNA samples isolated from blood and saliva.** Genomic DNAs isolated from PBMCs or saliva from the same four subjects were analyzed using the **(A)** 4qA BSS assay and 4qA-L BSS assay, and **(B)** the DUX4 5’ assay. Expected CpGs, based on predicted sequence composition of the unconverted region amplified, are listed in numerical order. Red boxes indicate methylated CpGs, blue boxes indicate unmethylated CpGs, and white boxes indicate no CpG at the expected site. The DNA methylation for the Q1 is indicated along with the range from the lowest percentage methylation to the highest percentage methylation in the set. *Neither the 4qA BS PCR nor the 4qA-L BS PCR produced a product from this subject, indicating that no 4qA or 4qA-L alleles were present; therefore, an alternative BSS protocol (4q/10q BSS) that amplifies both 4qA and 10qA alleles was performed (see Methods). The white boxes indicate no CpGs were detected at positions #16 and #55, which suggested these sequences were derived from 10qA. However, analysis of the complete BSS sequence data provided an additional non-CpG polymorphism that identified all sequences as being derived from 4C166H chromosomes.

A blind comparison of DNA methylation profiles using the three BSS protocols was performed on genomic DNAs isolated from blood and saliva from two clinically diagnosed and genetically confirmed FSHD1 subjects and two healthy first-degree relatives (Figure 4). The assays analyzed all 56 CpGs in the distal D4Z4 of each 4qA array, all 30 CpGs in the distal D4Z4 of 4qA-L linked arrays, when present, and 59 CpGs in the DUX4 5’ region of all samples, as described above (Figure 2). All FSHD subjects will possess at least one 4qA (or 4qA-L) allele, and non-FSHD control subjects have either two, one, or no 4qA (or 4qA-L) alleles. Healthy control subjects with either one or two 4qA/4qA-L alleles are predicted to show DNA hypermethylation (>35% methylation) on all assayed chromosomes, whereas those with 4qB/B genotypes will not produce a BS PCR product or in some rare instances produce a 10qA product that is effectively removed from analysis by identifying sequence polymorphisms. FSHD1 subjects must have at least one 4qA allele in *cis* with a contracted D4Z4. In FSHD1 subjects with 4qA/B haplotypes, all of the analyzed chromosomes are derived from the contracted D4Z4 array and are expected to show hypomethylation. In FSHD1 subjects with 4qA/A or 4qA-L/A-L haplotypes, on average half of the analyzed chromosomes will be derived from the contracted array and are expected to show DNA hypomethylation while the other half will be derived from the non-contracted array and are expected to show hypermethylation. In subjects with 4qA/A-L haplotypes, all of the BSS clones in each assay will be derived from the same chromosome, either contracted or non-contracted.

To avoid diluting the signature of FSHD1 by averaging with the methylation levels of the non-contracted array, we use the first quartile (Q1) of the methylation percent of all analyzed chromosomes as a summary statistic. This corresponds to dividing all sequences into two groups based on methylation percentage, and taking the median value of only those sequences in the lower group. If the total number of sequences is odd, there is the issue of whether to include the central sequence in the lower group or not before taking the median; to give it half weight we compute the median both ways, then take the arithmetic average. This corresponds to the R function quantiles with type = 5.

In a 4qA/A FSHD1 subject for whom all chromosomes with the contracted array have lower 4qA BSS methylation than any chromosomes with the non-contracted array, Q1 gives an estimate of the median 4qA methylation of just the contracted array. With n = 10 sequences analyzed, there is a 5.4% chance that more than 75% will arise from the non-contracted allele due to random sampling, so Q1 will not be an accurate reflection of the contracted allele; increasing n to 18 reduces the probability of this sort of failure to 1.5%.

Note, however, that if there is any overlap in methylation levels between alleles (as may be expected in healthy controls, FSHD2 subjects, and, potentially, in some FSHD1 subjects as well), then the half of analyzed sequences with lower methylation need not arise from a single allele, and Q1 underestimates the median methylation of any one allele. In the extreme case of no difference in methylation distributions between two 4qA alleles, or of 4qA/4qB genotypes (in which all sequences arise from a single allele), Q1 instead is an estimate of the lower quartile of methylation of one allele, rather than the median. This bias is tolerable for the present application, so for simplicity we use Q1 (Additional file 2: Table S1) as a summary statistic uniformly for all samples, without requiring the genotype to be known; we have also developed a mixture-model based statistical approach that aims to mitigate this bias [unpublished observations by Jones et al. 2014].

As shown in Figure 4A and Additional file 2: Table S1, the distal 4qA D4Z4 was dramatically hypomethylated in both blood and saliva samples for subjects 75194 (Q1 = 21.4% methylated, PBMCs; Q1 = 10.7% methylated, saliva) and 75204 (Q1 = 7.1% methylated, PBMCs; Q1 = 8.9% methylated, saliva), and was hypermethylated in both blood and saliva of subject 75195 (Q1 = 87.5% methylated, PBMCs; Q1 = 89.3% methylated, saliva). The 4qA-L BSS analysis indicated that the A-L haplotype was only present in subject 75194 and this allele was hypermethylated (Q1 = 70.8% methylated, PBMCs; Q1 = 80.0% methylated, saliva). Neither of these 4qA-specific BS PCRs produced a product from either the PBMCs or saliva of subject 75205, indicating that this subject lacked any 4qA alleles. Based on this analysis we predicted that subjects 75194 and 75204 were FSHD patients, and subjects 75195 and 75205 were healthy controls.

**Figure 5:**
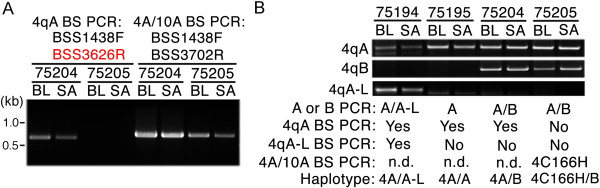
**PCR haplotyping. (A)** BS PCR products for subjects 75204 and 75205 using the 4qA DUX4 gene body primer set (left) or a primer set that non-specifically amplifies both 4qA and 10qA. BL: blood (PBMCs) and SA: saliva. **(B)** Genomic PCR amplification for either the 4qA or 4qB subtelomeres [15], as indicated. Although the 4qA D4Z4 gene body BS PCR did not produce a product for subject 75205 (No), standard PCR for 4qA alleles did produce a PCR product. These products were sequenced and confirmed as being 4C166H. These data together indicate that subject 75205 has a 4qB/C166H genotype. Additional predicted genotypes are indicated.

To further investigate the BSS results, we performed a second BS PCR on DNAs from subjects 75204 and 75205 utilizing a BS PCR primer set (primers BSS1438F and BSS3702R) that amplifies the distal D4Z4 region from both 4qA and 10qA for nested PCR (Figure 5A). The BSS profile of the 75205 products from both saliva and PBMCs showed no 4qA or 4qA-L chromosomes and suggested amplification of 10qA (Figure 4, 4A/10A row), as indicated by the lack of CpGs #16 and #55 (typically a 10A166 haplotype BSS signature). However, analysis of the entire amplified sequence revealed a polymorphism in all products that, when combined with the methyl-CpG profile, corresponded to the non-permissive 4C166H haplotype [18]. To confirm the haplotypes predicted by the BSS, genomic PCR was performed on all DNA samples to detect the presence of 4qA, 4qA-L, and 4qB subtelomeres (Figure 5B), as described [15]. As suggested by the BSS results in Figure 4A, subjects 75194, 75195, and 75204 all contained at least one 4qA allele and subject 75194 also contained one 4qA-L allele. Subjects 75204 and 75205 each tested positive for a 4qB allele. Interestingly, subject 75205 also tested positive for a 4qA allele from both PBMC and saliva DNAs despite producing no 4qA BS PCR product (Figure 5B), indicating that this 4qA haplotyping PCR also amplifies 4qC chromosomes. Sequence analysis of the genomic PCR products confirmed that subject 75205 has one chromosome with a 4C166H haplotype, consistent with the BSS data (Figure 4A), further supporting the specificity of the 4qA BSS assay. This more complete analysis supports our initial conclusions and provides additional information as follows: subjects 75194 (4qA/A-L) and 75204 (4qA/B) were FSHD patients and subjects 75195 (4qA/A) and 75205 (4qB/4C166H) were healthy controls.

BSS analysis of the DUX4 5’ promoter region is more complex (Figure 2). This analysis was designed to preferentially detect all 4q D4Z4s regardless of haplotype from both the contracted and non-contracted 4q chromosome arrays. Because in FSHD1 only the contracted chromosome 4 D4Z4 is hypomethylated [17], the observed proportion of hypomethylated sequences is expected to depend on the number of D4Z4 RUs in the contracted 4q array relative to the number of D4Z4 RUs on the non-contracted 4q array, together with chromosome 4q-type RUs on hybrid chromosome 10s, if present. In addition, preference for the 4q D4Z4 is based on a conserved 4q-specific polymorphism at the 3’ terminal base of a BSS PCR primer; however, since this relies on a single base polymorphism, there is the potential that a fraction of 10q-derived D4Z4 sequences could be amplified. In fact, sequence analysis of the DUX4 5’ BS PCR products identified both 4qA-specific polymorphisms and 10q-specific polymorphisms, indicating that although the reaction has a preference for 4q, it does not preclude amplification of some 10q array RUs. Fortunately, this does not adversely affect our analysis. For healthy controls, we anticipate that the vast majority of the analyzed chromosomes will show D4Z4 hypermethylation (>35% methylation) regardless of origin. By contrast, FSHD1 subjects should contain a combination of hypermethylated (from D4Z4 RUs residing in the non-contracted 4q array and both 10q arrays) and hypomethylated (from the D4Z4 RUs residing in the contracted 4q array) sequences with a clear minority of the analyzed D4Z4 RUs being hypomethylated; FSHD2 subjects should be hypomethylated (~ <25% methylation) on most sequences analyzed. Thus, the DUX4 5’ BSS assay is expected to be less sensitive than the 4qA and 4qA-L BSS assays in distinguishing FSHD1 from healthy controls; however, this assay should support those results, and would clearly distinguish FSHD2 from FSHD1 or healthy controls. Therefore, to more accurately distinguish FSHD1 from FSHD2 we use the mean percent methylation of each sample for comparison (Additional file 2: Table S1).

The DUX4 5’ BSS analysis was tested on the same eight genomic DNA samples as above (Figure 4B). As with the 4qA BSS assay, DUX4 5’ BS products from subjects 75195 (91.0% methylation mean, PBMCs; 94.4% methylation mean, saliva) and 75205 (71% methylation mean, PBMCs; 63.9% methylation mean, saliva) showed pronounced DNA hypermethylation in both PBMCs and saliva, suggesting that these two subjects were healthy controls. Subjects 75194 (47.8% methylation mean, PBMCs; 59.7% methylation mean, saliva) and 75204 (50.8% methylation mean, PBMCs; 59.3% methylation mean, saliva) showed less methylation than the putative controls but more methylation, on average, than found for these samples in the 4qA BSS analysis. However, in accordance with our predictions for FSHD1, these subjects contained a mixture of hypermethylated and hypomethylated DNA, resulting in a wide range of DNA methylation density per analyzed chromosome that reached much lower in subjects 75194 (5.1 to 78.0% methylation, PBMCs; 5.1 to 81.4% methylation, saliva) and 75204 (6.8 to 91.5% methylation, PBMCs; 5.1 to 88.1% methylation, saliva) compared with 75195 (72.9 to 100% methylation, PBMCs; 78.0 to 100% methylation, saliva) and 75205 (40.7 to 88.1% methylation, PBMCs; 32.2 to 86.4% methylation, saliva). This data indicates that subjects 75194 and 75204 are FSHD1 and not FSHD2 patients, while subjects 75195 and 75205 are healthy controls. In each case, the genomic DNAs isolated from PBMCs and saliva samples produced similar BSS results for each subject.

Upon final analysis, subjects 75194 and 75204 exhibited D4Z4 hypomethylation detected by the 4qA BSS analysis (Q1 < 25% methylated), indicative of FSHD, and by the DUX4 5’ BSS analysis they were clearly not FSHD2 (see below) and were thus predicted to be two FSHD1 patients. In fact, subjects 75194 and 75204 indeed had positive genetic tests for FSHD1. Importantly, subject 75204 (34 kb *Eco*RI/*Bln*I fragment corresponding to 9 D4Z4 RUs) and subject 75194 (27 kb *Eco*RI/*Bln*I fragment corresponding to 7 D4Z4 RUs) were both in the high end of the genetic FSHD1 contraction range, yet both were still accurately identified as FSHD1 by our analysis highlighting the sensitivity of these assays. Similarly, subjects 75195 and 75205, displaying hypermethylation at D4Z4 of all analyzed sequences by both the 4qA BSS and the DUX4 5’ BSS methods, were accurately determined to be healthy controls. With respect to the distal 4qA BSS analysis, subject 75195 was accurately identified from both blood and saliva genomic DNA as a healthy control, while control subject 75205 was accurately determined to lack a 4qA allele at either chromosome 4 (see below).

Overall, genomic DNAs isolated from blood and saliva provided similar epigenetic profiles of the FSHD-associated D4Z4 array in FSHD1 affected patients and healthy first-degree relatives. This test analysis confirmed the specificity of the 4qA BSS and 4qA-L BSS protocols for 4qA alleles over 10qA alleles or 4qB alleles. In addition, we have applied this analysis to myogenic cells or PBMCs from an additional 20 subjects having a clinical and genetic diagnosis of FSHD1 and 10 subjects confirmed as healthy unaffected. The simple cutoff of Q1 < 30% for 4qA and 4qA-L methylation accurately classified 19 of the 20 FSHD subjects and 9 of the 10 healthy controls (*P* = 7 × 10^-6^ by Fisher’s Exact Test); the one false positive was the only sample in the intermediate zone of 25% < Q1 < 35% [unpublished observations by Jones et al*.*, 2014]. We conclude that the described BSS analysis can readily identify FSHD1 hypomethylation, is suitable for epigenetic analysis of the D4Z4 array in both FSHD1 and healthy subjects, and that saliva samples are comparable to PBMCs in terms of providing suitable genomic DNA for DNA methylation analysis of the 4q35 D4Z4.

### Identification of the FSHD2 DNA hypomethylation signature

**Figure 6:**
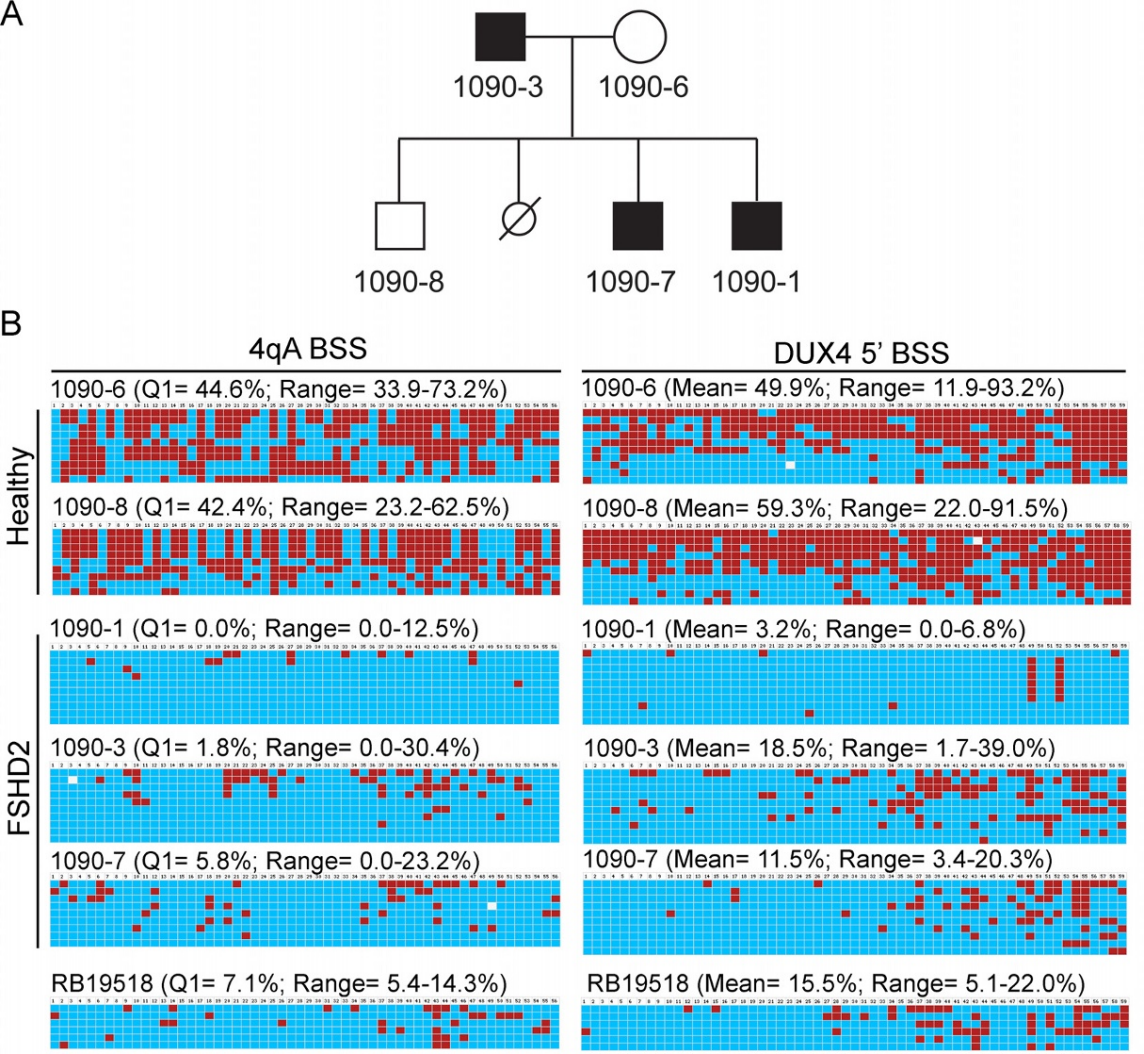
**BSS analysis of genomic DNA samples distinguishes FSHD2 from FSHD1. (A)** Partial pedigree for family 1090, which has a known FSHD2 mutation in the SMCHD1 gene that segregates with disease [27]. **(B)** The 4qA BSS analysis (left) and DUX4 5’ BSS analysis (right) for genomic DNAs isolated from subjects in family 1090 or subject RB19518, as indicated. Genomic DNAs were isolated from fibroblasts for subject 1090-1 and PBMCs for all other subjects. Expected CpGs, based on predicted sequence composition of the unconverted region amplified, are listed in numerical order. Red boxes indicate methylated CpGs, blue boxes indicate unmethylated CpGs, and white boxes indicate no CpG detected at the expected site. The Q1 percent methylation is indicated for the 4qA BSS assays and the mean percent methylation is indicated for the DUX4 5’ BSS assays.

Current genetic testing for FSHD, either by PGFE or molecular combing, detects a contracted 4qA D4Z4 array (FSHD1) and produces a negative result in ~5% of clinically diagnosed FSHD cases. These subjects are candidates for FSHD2. FSHD2 can be diagnosed in two ways: genomic sequencing of the *SMCHD1* gene for a known (or likely) FSHD2 mutation (valid for ~85% of cases) or epigenetic analysis of the D4Z4 array (valid for 100% of known cases). The distinguishing feature of FSHD2 is DNA hypomethylation (<25% methylation) of both the 4q35 and 10q26 D4Z4 arrays [19, 21]. In addition, as is the case with FSHD1, FSHD2 requires at least one permissive 4qA allele. Since our BSS analysis identifies 4qA haplotypes and determines the DNA methylation profiles of the D4Z4 arrays on both 4q chromosomes, we sought to determine if our method could be used to identify cases of FSHD2. We used genomic DNAs isolated from fibroblasts or blood obtained from a family containing three known FSHD2 subjects possessing a mutation in *SMCHD1* and two unaffected relatives (Figure 6) [27]. Our BSS analysis of the DUX4 5’ region showed extreme DNA hypomethylation (3.2%, 18.5%, and 11.5% methylation means) in all three FSHD2 subjects and, conversely, DNA hypermethylation (49.9% and 59.3% methylation means) of both healthy controls (Figure 6B, right column). The 4qA BSS analysis positively detected at least one 4qA allele in each FSHD2 subject with concurrent DNA hypomethylation of all analyzed sequences, and healthy controls were hypermethylated on all 4qA chromosomes (Figure 6B, left column). These DNA methylation profiles are strikingly distinct from those found for FSHD1 (Figure 4) and clearly identify these subjects as FSHD2. We conclude that our BSS assay can be used to positively detect an FSHD2 epigenetic signature with a permissive 4A subtelomere, readily distinguishable from that of FSHD1 or healthy controls, using standard genomic DNA preparations from multiple sources.

We further tested the utility of this assay by analyzing PBMC genomic DNA isolated from a subject (RB19518) who was clinically diagnosed with FSHD but had a negative genetic test result for FSHD1 by the standard PFGE technique. FSHD2 is characterized by <25% methylation of all four 4q and 10q D4Z4 arrays. In less than five days following retrieval of genomic DNA, the results of our FSHD BSS assays showed a 15.5% methylation mean in the DUX4 5’ region, with a range of 5.1 to 22% methylation, and a Q1 = 7.1% methylation using the 4qA BSS assay, with a range of 5.4 to 14.3% methylation, indicating that all detected D4Z4s were hypomethylated (Figure 6B, lower panels). This analysis indicated that this subject had a clear FSHD2 epigenetic signature and a likely permissive 4A subtelomere and thus, when combined with the clinical evaluation, is very likely FSHD2. We conclude that this assay is a quick and efficient way to determine FSHD2 epigenetic signatures and does not require HMW DNA.

### Identification and elimination of the rare 10A176T and 4A166 non-permissive haplotypes from BSS analysis

It is important to keep in mind that the majority of analyzed chromosomes in FSHD and healthy subjects will have chromosomes with standard 4qA (44%, including 4qA-L), 4qB (50%), and 10qA (91%) haplotypes; however, there are some important exceptions to consider [18]. Two of them are the rare, non-permissive 10A176T and 4A166 haplotypes, neither of which is identified by current standard diagnostic testing [18]. Since D4Z4 arrays of 10A176T have chromosome 4-like resistance to digestion with *Bln*-I, the enzyme used to distinguish chromosome 4 arrays from chromosome 10 arrays, this chromosome 10 haplotype can be misidentified as chromosome 4 by PFGE analysis and 4A166 linked arrays are indistinguishable from permissive 4qA arrays using PFGE. Thus, the presence of 10A176T or 4A166 can complicate genetic diagnosis and epigenetic analyses, particularly when these haplotypes are associated with a short D4Z4 array. Since the prevalence of 10A176T and 4A166 in the European population are ~2.5% and ~4.1%, respectively, it is to be expected that ~1 out of 15 FSHD patients, healthy control subjects, and even patients with other myopathies will carry one of these potentially confusing haplotypes [18]. Fortunately, the 10A176T and 4A166 alleles have several distinguishing polymorphisms and can be identified by PCR haplotyping of genomic DNA [15]. However, for our diagnostic purposes as well as epigenetic analyses, it is important to know if our 4qA and 4qA-L BSS assays can identify and/or eliminate these non-permissive 10A176T or 4A166 haplotypes from the BSS analysis.

**Figure 7:**
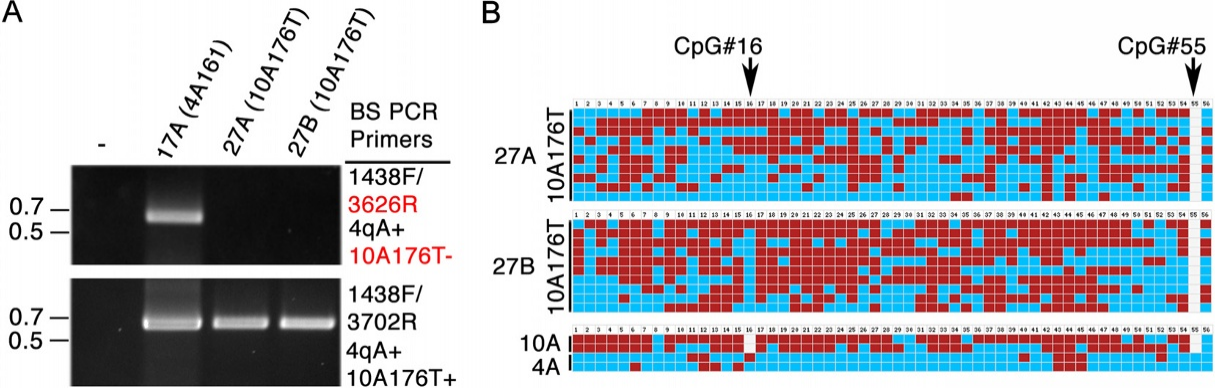
**The 4qA BSS analysis does not amplify from 10A176T or 4A166 alleles. (A)** The 4qA BSS assay (upper panel) is specific for 4qA sequences (present in sample 17A) and does not amplify the non-permissive 10A176T or 4A166 alleles present in samples 27A and 27B. BSS PCR using oligonucleotide primers that do not distinguish between 4A and 10A176T (lower panel) amplifies robustly from all three samples. **(B)** Sequence analysis of the products from samples 27A and 27B confirmed their origins as being from a 10A176T allele. The lack of a detectable CpG at position #55 but the presence of a CpG at position #16 identifies these as derived from a chromosome with a 10A176T haplotype. Expected CpGs, based on predicted sequence composition of the unconverted region amplified, are listed in numerical order. Red boxes indicate methylated CpGs, blue boxes indicate unmethylated CpGs, and white boxes indicate no CpG detected at the expected site.

Therefore, we tested our 4qA and 4qA-L BSS assays on genomic DNAs known to contain the 10A176T allele. We identified two subjects (27A and 27B) from the same family who have very short D4Z4 arrays in *cis* with the 10A176T haplotype and lacking a permissive 4qA allele [6]. As shown (Figure 7A, upper panel), no BS PCR product was amplified from these subjects using these assays. This was not surprising considering both 4A166 and 10A176T share the same sequence polymorphisms in the primer BSS3626R that was used to eliminate BS PCR product amplification from non-permissive 10qA (Additional file 1: Figure S1). To confirm the content and integrity of these BS-converted DNAs, we used an alternative BSS primer that is not predicted to distinguish 4A from 10A176T for amplification (Figure 7A, lower panel). Analysis of the amplified product revealed that all sequences matched the predicted polymorphisms for 10A176T, and not 4A or 10A, including the lack of CpG #55 but not CpG #16 (Figure 7B). Therefore, this additional BSS assay can be used to both positively identify and study the methylation status of chromosomes with the 10A176T haplotype. We conclude that the 4qA and 4qA-L BSS assays do not amplify the 10A176T or 4A166 haplotypes and effectively eliminate them from the methylation analysis.

### Combined analysis and epigenetic diagnosis of FSHD

**Figure 8:**
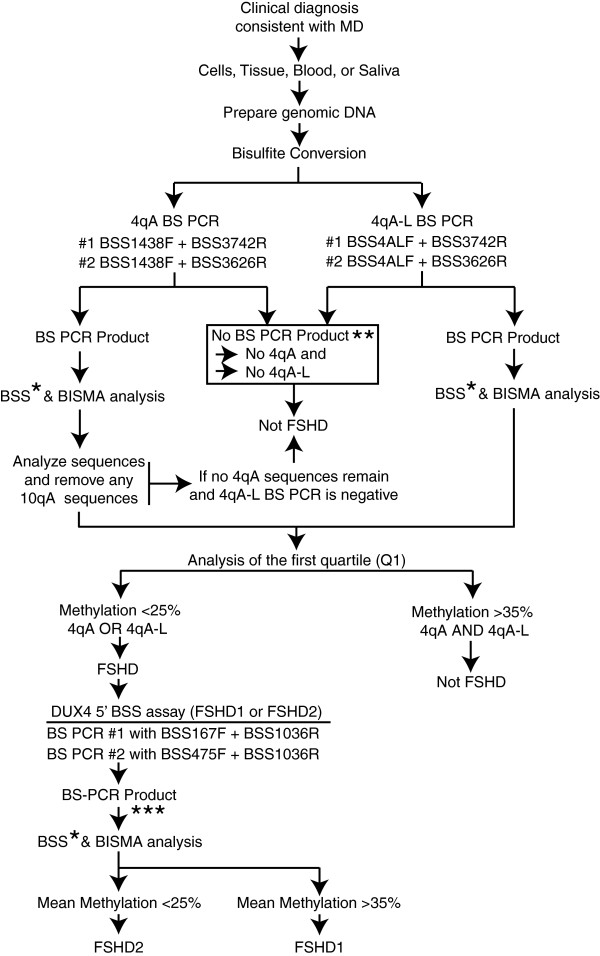
**Flow chart of epigenetic diagnosis of FSHD1 and FSHD2 by BSS.** Clinical samples, including saliva, blood, muscle tissue, or cells, from patients with a clinical diagnosis of neuromuscular disease consistent with FSHD can be used for genomic DNA isolation and an epigenetic diagnosis of FSHD1 or FSHD2. The first level BSS assays, namely the 4qA and 4qA-L BSS assays, identify FSHD. The second level assay, namely the DUX4 5’ assay, distinguishes between FSHD1 and FSHD2. *Sequence analysis can be performed by subcloning and Sanger sequencing of a minimum of 10 independent clones; alternatively, a NGS approach can be used. Sequences are screened for 10A, 10A176T, and 4A166 and, if present, those sequences are removed from the analysis. The lower quartile (Q1) of the percent methylation is computed for the remaining sequences, to improve sensitivity for detecting hypomethylation on a contracted allele when roughly half the sequences are from a non-contracted allele and are hypermethylated. **If no BS PCR product is generated then the subject likely lacks a permissive 4A haplotype. Genomic PCRs for A- and B-type subtelomeres and sequencing can be used to confirm the results. ***Sequence analysis of the BS PCR product, which is derived from both 4q and 10q arrays and thus present in all samples, can be performed by subcloning and Sanger sequencing of a minimum of 10 independent clones; alternatively, a NGS approach can be used. The mean DNA methylation of 10 sequences is not expected to identify strong changes in FSHD1 patients since the vast majority of sequences are likely derived from either the non-contracted 4q or either of the 10q D4Z4 arrays; however, FSHD2 shows hypomethylation (<25% methylation mean) on both 4q and both 10q D4Z4 arrays. Precise cutoffs may need to be adjusted as more samples are examined.

The three BSS assays presented use DNA methylation levels of the terminal D4Z4 RU to distinguish FSHD from healthy unaffected subjects as well as FSHD1 from FSHD2 (Figure 8). However, in describing the BSS methods here, only two FSHD1 subjects, four FSHD2 subjects, and four unaffected control subjects were used for this proof-of-principle analysis (Figures 4 and 6). To confirm that the epigenetic signatures of the distal 4qA and DUX4 5’ regions could truly be used in the diagnosis of FSHD, we analyzed data produced from our much more extensive epigenetic study of FSHD1-affected and FSHD1-non-manifesting subjects, which applied this protocol to a larger number of samples [unpublished observations by Jones et al*.*, 2014]. PBMCs or myogenic cells from a total of 20 clinically affected FSHD1 and 10 healthy subjects, all confirmed by PFGE as FSHD1 or unaffected, were analyzed. The FSHD1 contractions ranged from 14 to 32 kb *Eco*RI/*Bln*I fragments in *cis* with a permissive A subtelomere, while the shortest 4qA allele *Eco*RI/*Bln*I fragment from all unaffected healthy controls was >53 kb. Our analysis of DNA methylation using the 4qA BSS assay with cutoff of Q1 < 30% accurately classified 19 of the 20 FSHD subjects and 9 of the 10 healthy controls. Interestingly, our previous analysis of DUX4 expression showed that myogenic cells from the false positive, sample 16U, express *DUX4-fl* mRNA and protein [6], consistent with our epigenetic analysis. This is in stark contrast to the recent BSS method for FSHD published by Gaillard et al. [34], which reported significant population differences between FSHD1 and healthy subjects, but has limited diagnostic benefit on an individual basis. This is not surprising considering the authors use an approach that assays all D4Z4 repeat units from chromosome 4 and chromosome 10 (and perhaps other D4Z4 repeats as well, given the large number of polymorphisms observed in CpG sites [30]), since sequences from the contracted 4q allele then make a small and highly variable contribution to the overall average methylation level. Methylation levels for control samples showed a coefficient of variation (SD/mean) of ~15% in Figure 5C (left) by Gaillard et al. [34]; thus if only ~10% of sequences in an FSHD1 sample are derived from the contracted allele (as would be expected with, for example, 5 D4Z4 RU on the contracted 4q allele and 45 D4Z4 RU on the non-contracted 4q allele, a conservative estimate as it ignores D4Z4 repeats on other chromosomes), their impact on the observed average methylation level is less than the normal variation between control subjects.

Even a small false positive rate (e.g., 1%) can result in poor positive predictive value when applied to populations in which FSHD prevalence is smaller still (such as the general population). However, since individuals with a variety of non-FSHD muscular dystrophies have D4Z4 methylation-levels similar to healthy controls [17], our assay can be used as a differential diagnostic between FSHD and other diseases when applied to patients with clinical characteristics consistent with FSHD. In addition, all of the samples from FSHD1 subjects that were tested with the DUX4 5’ BSS assay showed DNA methylation levels above 25%, consistent with an FSHD1 diagnosis and not FSHD2. Conversely, all FSHD2 subjects showed DNA methylation levels well below 25% in both the DUX4 5’ and 4qA BSS assays, providing clear evidence for FSHD2 as opposed to FSHD1. However, while this assay is specific for the generally FSHD permissive 4qA allele, as with standard FSHD1 testing by PFGE or molecular combing [24], it does not positively identify a functional *DUX4* PAS, which is required of a truly permissive 4qA allele. We conclude that the combination of these two assays used for individuals with clinical symptoms of FSHD is diagnostic for FSHD1 and FSHD2 (Figure 6).

## Conclusions

We have developed a PCR-based technique to identify and distinguish all forms of FSHD from DNA methylation profiles in blood, saliva, or fibroblasts. The combination of two BSS assays allows the analysis of the DNA methylation profile of a portion of the distal 4q35 D4Z4 RU associated with all forms of FSHD. These assays are specific for 4q chromosomes with the FSHD-associated A-type subtelomere and do not amplify D4Z4 sequence from B-type subtelomeres. Sequences from non-permissive 10qA (including 10qA176T) and 4A166 are not amplified in most assays and, if present (a sign of PCR primer degradation), are readily removed from analysis. The DNA methylation profiles produced by this assay clearly distinguish between FSHD and healthy subjects (Figure 8). We also describe a companion BSS assay that analyzes the DNA methylation status of a region 5’ of the *DUX4* gene that is present on all 4q35 and 10q26 D4Z4 repeats. Utilizing the three BSS assays in combination discloses the DNA methylation status of the distal D4Z4 in the context of overall 4q35 D4Z4 DNA methylation. Therefore, in addition to determining contracted 4qA-specific DNA hypomethylation characteristic of FSHD1 and overall D4Z4 hypermethylation in healthy controls, this assay identifies FSHD2-specific DNA hypomethylation signatures on the 4qA allele and clearly distinguishes them from FSHD1 signatures (Figure 8). Importantly, this analysis does not require HMW genomic DNA and can be performed on genomic DNAs isolated from blood or saliva, producing similar results. Additionally, the protocols can readily be modified with bar-coded oligonucleotide primers such that data acquisition and analysis can be performed using next-generation sequencing technology.

## Methods

### Subjects and methods

The University of Massachusetts Medical School Institutional Review Board approved this study; participants provided written informed consent. Patients 75194, 75204, and RB19518 were clinically diagnosed as FSHD. Patients 75194 and 75204 each had a positive genetic test for FSHD1 and RB19518 had a negative genetic test for FSHD1. Subjects 75205 (healthy relative of 75204) and 75195 (healthy relative of 75194) were clinically unaffected. The FSHD2 family cohort (1090) was previously described [27] and contains a mutation in the *SMCHD1* gene that segregates with disease. Myogenic cells for cohort 27 were obtained from the previously described Wellstone Center cell repository housed at the University of Massachusetts Medical School [6, 35].

### Sample collection and DNA preparation

Saliva samples (2 mL) were collected from subjects using the DNAgenotek Oragene Discover (ORG-500) DNA collection kit and genomic DNAs were isolated using the manufacturer’s recommended protocol. Genomic DNAs from blood samples were isolated using the Qiagen Puregene DNA isolation kit using the recommended protocol.

### DNA methylation analysis

DNA methylation was analyzed by BSS assay. BS conversion was performed on 1 μg of genomic DNA using the EpiTect Bisulfite Kit (Qiagen) as per the manufacturer’s instructions, and 200 ng of converted genomic DNA was used per PCR. For the 4qA BSS analysis, converted DNA was amplified by nested PCR using oligonucleotide primers and thermocycling conditions that amplify 4qA but not 4qB; the initial PCR was performed with oligonucleotide primers BSS1438F (5’-GTTTTGTTGGAGGAGTTTTAGGA) and BSS3742R (5’-AACATTCAACCAAAATTTCACRAAA) and then followed by nested PCR with oligonucleotide primers BSS1438F and BSS3626R (5’-AACAAAAATATACTTTTAACCRCCAAAAA) using 10% of the first PCR product as template. Polymorphic nucleotide changes that preferentially amplify the 4A subtelomeric region are underlined. The BSS3742R sequence does not exist in 4B or 10B and utilizes a polymorphic change at bp 7946 in FJ439133 to eliminate 10A166, and BSS3626R utilizes polymorphic changes at bp 7827 in FJ439133 to eliminate 10A, 4B, and 10B [15]. All PCRs were performed using GoTaq Hot Start Polymerase (Promega) as follows: 94°C for 2 min, 25 cycles of 94°C for 15 sec, 58°C for 20 sec, and 72°C for 50 sec, followed by a final extension at 72°C for 10 min. The 593-bp PCR product spans the end of full-length DUX4 exon 1 to the beginning of DUX4 exon 3, therefore allowing specific analysis of the methylation status of the most distal 4qA D4Z4 repeat, which contains 57 CpGs (Additional file 1: Figure S1A). For the 4qA-L BSS analysis, converted DNA was similarly amplified by nested PCR. The initial PCR was performed with oligonucleotide primers BSS4qALF (5’-TTATTTATGAAGGGGTGGAGTTTGTT) and BSS3742R, and then followed by nested PCR with oligonucleotide primers 4qALF and BSS3626R using 10% of the first PCR product as template. All PCRs were performed using GoTaq Hot Start Polymerase (Promega) as follows: 94°C for 2 min, 25 cycles of 94°C for 15 sec, 58°C for 20 sec, and 72°C for 30 sec followed by a final extension at 72°C for 10 min. The 354-bp PCR product spans the 3’ end of the extended 4qA-L D4Z4 repeat to the beginning of DUX4 exon 3, therefore allowing specific analysis of the methylation status of the most distal 4qA D4Z4 repeat sequence, which contains 30 CpGs (Additional file 1: Figure S1D). When no PCR product was obtained with either the 4qA- or 4qA-L-specific BS PCRs, DNA methylation status of same distal D4Z4 region was analyzed using primer BSS3702R (5’-AAAACCAACRAACTCCCTTACAC) instead of BSS3626R. BSS3702R amplifies distal D4Z4 from both 10A and 4A. For the DUX4 5’ region, BS-converted DNA was amplified by nested PCR as described above. The initial PCR was performed with oligonucleotide primers BSS167F (5’-TTTTGGGTTGGGTGGAGATTTT) and BSS1036R (5’-AACACCRTACCRAACTTACACCCTT), and then followed by nested PCR with oligonucleotide primers BSS475F (5’-TTAGGAGGGAGGGAGGGAGGTAG) and BSS1036R. A polymorphic nucleotide change at bp 6748 in FJ439133 (underlined) was used to preferentially amplify the 4A subtelomeric region. This 578-bp PCR product contains 61 CpGs to preferentially analyze the methylation status of the DUX4 5’ region of chromosome 4-type D4Z4 repeats (Additional file 1: Figure S1E).

All BS PCR products were cloned into the pGEM-T Easy Vector system I (Promega) for sequencing analysis. At least 10 clones were sequenced for each subject and their methylation status was analyzed using web-based analysis software BISMA (http://biochem.jacobs-university.de/BDPC/BISMA/) [36] with the default parameters. Default parameters have a lower threshold of 90% identity to the reference sequence, a lower threshold of BS conversion rate of 95%, and remove identical sequences derived from the same genomic template based on conversion artifacts. To remove PCR amplification bias, 1 CpG in BSS3626R primer and 2 CpGs in BSS1036R primer were removed from the analysis; therefore, a total of 56 CpGs, 30 CpGs, and 59 CpGs were analyzed for the 4qA, 4qA-L, and DUX4 5’ region, respectively. The “R” designation in primer sequences represents a purine (A or G).

### Detection of 10A176T haplotype

BSS analysis using our 4qA-specific BSS primers and conditions does not amplify 10A176T alleles and will eliminate 10A176T from analysis. To confirm a 10A176T haplotype or analyze its DNA methylation status, oligonucleotide primer BSS3626R was replaced with BSS3702R. The bases corresponding to the 55th CpG in the 4qA BSS fragment are “TA” in 10A176T alleles due to the G7820A polymorphic change, and the C7808A polymorphism can be identified as an “A” instead of a “T” at this position in the BS-converted 10A176T [15].

### Detailed genotyping of 4q chromosomes

Standard genomic PCR was performed on non-converted DNA to identify the 4qA, 4qA-L, and 4qB chromosomes as previously described [15].

## Electronic supplementary material

Additional file 1: Figure S1: BSS products. **(A)** The 4qA BS-converted PCR product is shown. BSS primer sequences are highlighted in orange (forward) or blue (reverse). Base pair changes in the BS-converted sequence between the permissive 4A and non-permissive 4A, 10A, and 10B haplotypes are highlighted in red (permissive) and yellow (non-permissive). The CpG dinucleotides that would be missing from the analysis in the designated haplotypes are identified by number and are underlined. Y = C or T. **(B)** 4qA BS PCR primers that have undergone freeze-thaw several times produce minor PCR products (*), using DNA from cells lacking permissive 4qA alleles. None of these products correspond to 4qA or 4qB and occasionally correspond to 10qA. **(C)** Output analysis from BISMA comparing a typical 4qA BSS analysis with the rare non-permissive 10A166 or 4A166 haplotype BSS outputs that may appear, as in B, above. These are readily recognized by the absence of CpGs #16 and 55 (black arrows) and eliminated from analysis. **(D)** The 4qA-LBS-converted PCR product is shown. BSS primers are highlighted in orange (forward) or blue (reverse). Base pair changes between 4A-L and non-permissive 4A and 10A haplotypes are highlighted in red (permissive) and yellow (non-permissive). **(E)** The DUX4 5’ BS-converted PCR product. BSS primers are highlighted in orange (forward) or blue (reverse), with the 4q-specific D4Z4 polymorphism in highlighted in red and the 10q D4Z4 polymorphism highlighted in yellow. (PDF 725 KB)

Additional file 2: Table S1: BSS assay DNA methylation data. (PDF 48 KB)

## Abbreviations

BS PCR: Bisulfite PCR
BSS: Bisulfite sequencing
FSHD: Facioscapulohumeral muscular dystrophy
HMW: High molecular weight
PAS: Polyadenylation signal
PBMCs: Peripheral blood mononuclear cells
PCR: Polymerase chain reaction
PFGE: Pulse-field gel electrophoresis
Q1: First quartile
RU: Repeat unit.
