# Supplementary material for: Identifying diagnostic DNA methylation profiles for facioscapulohumeral muscular dystrophy in blood and saliva using bisulfite sequencing
Source: Clin Epigenetics. 2014 Oct 29;6(1):23. doi: 10.1186/1868-7083-6-23 (PMC4232706; doi:10.1186/1868-7083-6-23)
Supplement: Supplementary file 1 — Additional file 1: Figure S1: BSS products. (A) The 4qA BS-converted PCR product is shown. BSS primer sequences are highlighted in orange (forward) or blue (reverse). Base pair changes in the BS-converted sequence between the permissive 4A and non-permissive 4A, 10A, and 10B haplotypes are highlighted in red (permissive) and yellow (non-permissive). The CpG dinucleotides that would be missing from the analysis in the designated haplotypes are identified by number and are underlined. Y = C or T. (B) 4qA BS PCR primers that have undergone freeze-thaw several times produce minor PCR products (*), using DNA from cells lacking permissive 4qA alleles. None of these products correspond to 4qA or 4qB and occasionally correspond to 10qA. (C) Output analysis from BISMA comparing a typical 4qA BSS analysis with the rare non-permissive 10A166 or 4A166 haplotype BSS outputs that may appear, as in B, above. These are readily recognized by the absence of CpGs #16 and 55 (black arrows) and eliminated from analysis. (D) The 4qA-LBS-converted PCR product is shown. BSS primers are highlighted in orange (forward) or blue (reverse). Base pair changes between 4A-L and non-permissive 4A and 10A haplotypes are highlighted in red (permissive) and yellow (non-permissive). (E) The DUX4 5’ BS-converted PCR product. BSS primers are highlighted in orange (forward) or blue (reverse), with the 4q-specific D4Z4 polymorphism in highlighted in red and the 10q D4Z4 polymorphism highlighted in yellow. (PDF 725 KB) [file 13148_2014_88_MOESM1_ESM.pdf]

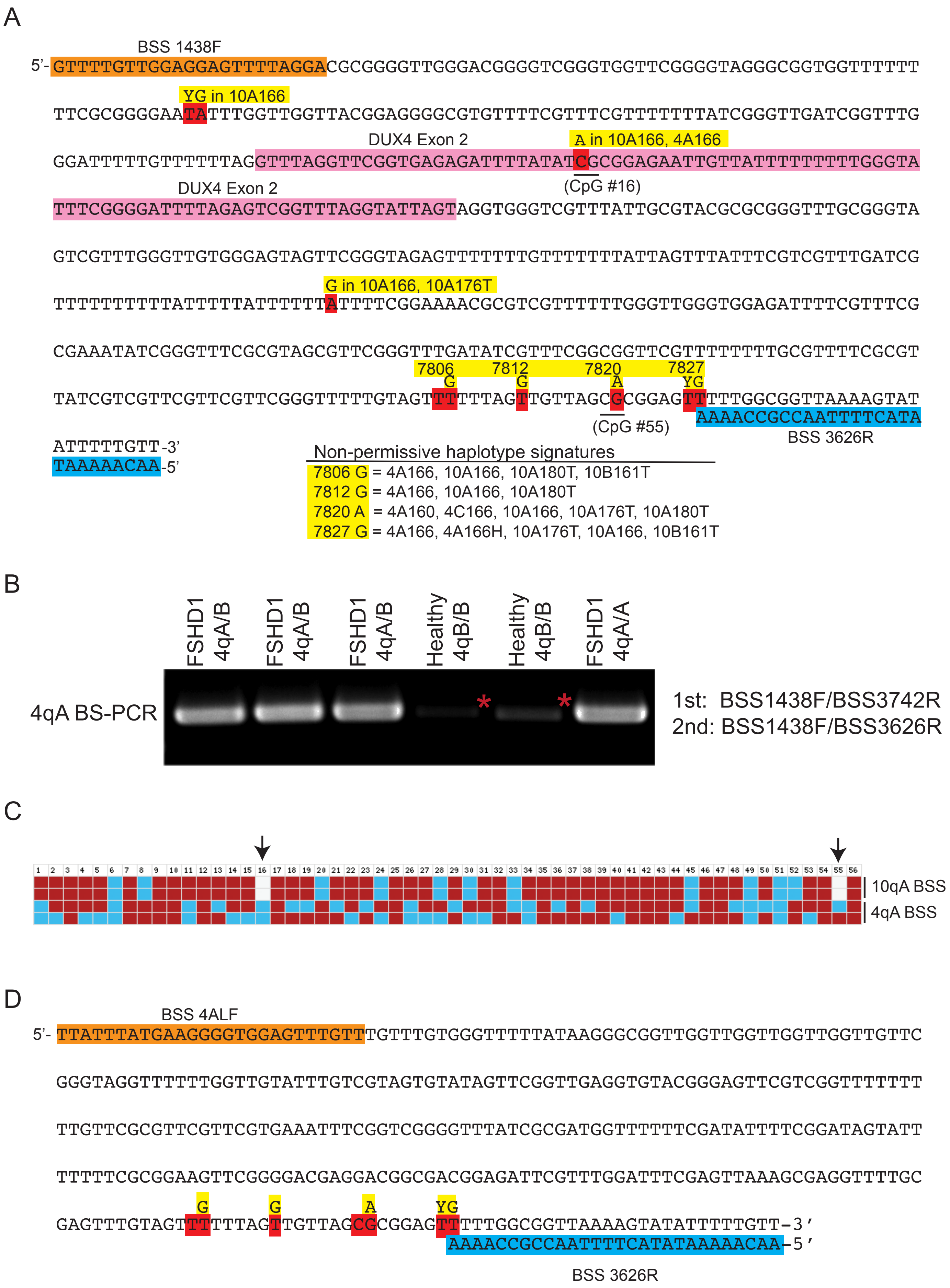
A
BSS 1438F
5'- GTTTTGTTGGAGGAGTTTTAGGACGCGGGGTTGGGACGGGGTCGGGTGGTTCGGGGTAGGGCGGTGGTTTTTT
YG in 10A166
TTCGCGGGGAATATTTGGTTGGTTACGGAGGGGCGTGTTTTCGTTTCGTTTTTTTATCGGGTTGATCGGTTTG
DUX4 Exon 2
A in 10A166, 4A166
GGATTTTTGTTTTTTAGGTTTAGGTTCGGTGAGAGATTTTATATCGCGGAGAATTGTTATTTTTTTTTGGGTA
(CpG #16)
DUX4 Exon 2
TTTCGGGGATTTTAGAGTCGGTTTAGGTATTAGTAGGTGGGTCGTTTATTGCGTACGCGCGGGTTTGCGGGTA
GTCGTTTGGGTTGTGGGAGTAGTTCGGGTAGAGTTTTTTTGTTTTTTTATTAGTTTATTTCGTCGTTTGATCG
G in 10A166, 10A176T
TTTTTTTTTTATTTTTATTTTTTATTTTCGGAAAACGCGTCGTTTTTTGGGTTGGGTGGAGATTTTCGTTTCG
CGAAATATCGGGTTTCGCGTAGCGTTCGGGTTTGATATCGTTTCGGCGGTTCGTTTTTTTTGCGTTTTCGCGT
7806 7812 7820 7827
G G A YG
TATCGTCGTTCGTTCGTTCGGGTTTTTGTAGTTTTTTAGTTGTTAGCGCGGAGTTTTTGGCGGTTAAAAGTAT
AAAACCGCCAATTTTCATA
(CpG #55)
BSS 3626R
ATTTTTGTT-3'
TAAAAACAA-5'
Non-permissive haplotype signatures
7806 G = 4A166, 10A166, 10A180T, 10B161T
7812 G = 4A166, 10A166, 10A180T
7820 A = 4A160, 4C166, 10A166, 10A176T, 10A180T
7827 G = 4A166, 4A166H, 10A176T, 10A166, 10B161T
B
FSHD1 4qA/B
FSHD1 4qA/B
FSHD1 4qA/B
Healthy 4qB/B
Healthy 4qB/B
FSHD1 4qA/A
4qA BS-PCR
*
*
1st: BSS1438F/BSS3742R
2nd: BSS1438F/BSS3626R
C
1 2 3 4 5 6 7 8 9 10 11 12 13 14 15 16 17 18 19 20 21 22 23 24 25 26 27 28 29 30 31 32 33 34 35 36 37 38 39 40 41 42 43 44 45 46 47 48 49 50 51 52 53 54 55 56
10qA BSS
4qA BSS
D
BSS 4ALF
5'- TTATTTATGAAGGGGTGGAGTTTGTTTGTTTGTGGGTTTTTATAAGGGCGGTTGGTTGGTTGGTTGGTTGTTC
GGGTAGGTTTTTTGGTTGTATTTGTCGTAGTGTATAGTTCGGTTGAGGTGTACGGGAGTTCGTCGGTTTTTTT
TTGTTCGCGTTCGTTCGTGAAATTTCGGTCGGGGTTTATCGCGATGGTTTTTTCGATATTTTCGGATAGTATT
TTTTTCGCGGAAGTTCGGGGACGAGGACGGCGACGGAGATTCGTTTGGATTTCGAGTTAAAGCGAGGTTTTGC
G G A YG
GAGTTTGTAGTTTTTTAGTTGTTAGCGCGGAGTTTTTGGCGGTTAAAAGTATATTTTTGTT-3'
AAAACCGCCAATTTTCATATAAAAACAA-5'
BSS 3626R

E

BSS +475

5'-TTAGGAGGGAGGGAGGGAGGTAGGGAGGTAGGGAGGAACGGAGGGAAAGATAGAGCGACGTAGGGATTGGGGG

CGGGCGGGAGGGAGTCGGGGACGGGGGGAGGAAGGTAGGGAGGAAAAGCGGTTTTCGGTTTTCGGGAGTAGCG

GGATTTTCGTTTTTCGGGAAAACGGTTAGCGTTCGGCGCGGGTTGAGGGTTGGGTTTATAGTCGTCGCGTCGG

TCGGCGGGGTATTATTTATTCGTTTCGGTTTCGTGGTTTAGGGAGTGGGCGGTTTTTTTCGGGATAAAAGATC

GGGATTCGGGTTGTCGTCGGGTTTTTATTCGCGCGGTTTATAGATCGTATATTTTTAGGTTGAGTTTTGTAAC

GCGGCGCGAGGTCGATAGTTTCGGTTACGGAGGAGTTATACGTAGGACGACGGAGGCGTGATTTTGGTTTTCG

CGTGGTTTTGTTTTTCGTAAGGCGGTTTGTTGTTTACGTTTTTTCGGTTTTCGAAAGGTTGGTTATGTCGATT

G in 10q D4Z4

GTTTGTTTTCGGAGTTTTGCGGGTATTCGGAAATATGTAGGGAAGGGTGTAAGTTCGGTACGGTGTT -3'

TTCCCACATTCAAGCCATGCCACAA -5'

BSS -1036

Figure S1: BSS products. A) The 4qA BS-converted PCR product is shown. BSS primer sequences are highlighted in orange (forward) or blue (reverse). Base pair changes in the BS-converted sequence between the permissive 4A and nonpermissive 4A, 10A, and 10B haplotypes are highlighted in red (permissive) and yellow (nonpermissive). The CpG dinucleotides that would be missing from the analysis in the designated haplotypes are identified by number and are underlined. Y= C or T. B) 4qA BS PCR primers that have undergone freeze-thaw several times produce minor PCR products (*), using DNA from cells lacking permissive 4qA alleles. None of these products correspond to 4qA or 4qB and occassionally correspond to 10qA. C) Output analysis from BISMA comparing a typical 4qA BSS abakysis with the rare nonpermissive 10A166 or 4A166 haplotype BSS outputs that may appear, as in B, above. These are readily recognized by the absence of CpGs #16 and 55 (black arrows) and eliminated from analysis. D) The 4qA-L BS-converted PCR product is shown. BSS primers are highlighted in orange (forward) or blue (reverse). Base pair changes between 4A -L and nonpermissive 4A and 10A haplotypes are highlighted in red (permissive) and yellow (nonpermissive). E) The DUX4 5' BS-converted PCR product. BSS primers are highlighted in orange (forward) or blue (reverse), with the 4q-specific D4Z4 polymorphism in highlighted in red and the 10q D4Z4 polymnorphism highlighted in yellow.
