# Supplementary material for: Identifying diagnostic DNA methylation profiles for facioscapulohumeral muscular dystrophy in blood and saliva using bisulfite sequencing
Source: Clin Epigenetics. 2014 Oct 29;6(1):23. doi: 10.1186/1868-7083-6-23 (PMC4232706; doi:10.1186/1868-7083-6-23)
Supplement: Supplementary file 2 — Additional file 2: Table S1: BSS assay DNA methylation data. (PDF 48 KB) [file 13148_2014_88_MOESM2_ESM.pdf]

Table S1: BSS assay DNA methylation data

Subject	Cells	BSS.assay	num.seqs	mean	min	Q1	median	Q3	max
PT-1090-1	HDF	4qA	10	2.7	0	0	0.9	1.8	12.5
PT-1090-1	HDF	DUX4 5'	10	3.2	0	3.4	3.4	3.4	6.8
PT-1090-3	PBMC	4qA	10	10.4	0	1.8	4.5	17.9	30.4
PT-1090-3	PBMC	DUX4 5'	10	18.5	1.7	11.9	20.3	23.7	39
CTL-1090-6	PBMC	4qA	10	53.6	33.9	44.6	52.7	57.1	73.2
CTL-1090-6	PBMC	DUX4 5'	10	49.9	11.9	19	53.4	76.3	93.2
PT-1090-7	PBMC	4qA	9	9.1	0	5.8	8.9	12.5	23.2
PT-1090-7	PBMC	DUX4 5'	10	11.5	3.4	6.8	11.9	15.3	20.3
CTL-1090-8	PBMC	4qA	9	51.6	23.2	42.4	60.7	62.5	62.5
CTL-1090-8	PBMC	DUX4 5'	10	59.3	22	27.1	66.9	84.7	91.5
75194	PBMC	4qA	10	31.1	5.4	21.4	26.8	33.9	64.3
75194	Saliva	4qA	10	17.6	1.8	10.7	17.9	25.5	32.1
75194	PBMC	4qA-L	19	80.7	56.7	70.8	83.3	90	93.3
75194	Saliva	4qA-L	14	83.6	70	80	83.3	86.7	96.7
75194	PBMC	DUX4 5'	11	47.8	5.1	24.2	64.4	71.2	78
75194	Saliva	DUX4 5'	10	59.7	5.1	50.8	66.9	72.9	81.4
75195	PBMC	4qA	10	91	82.1	87.5	91	94.6	98.2
75195	Saliva	4qA	10	90.5	82.1	89.3	89.3	94.6	98.2
75195	PBMC	DUX4 5'	10	94.2	72.9	93.2	96.6	98.3	100
75195	Saliva	DUX4 5'	10	94.4	78	93.2	95.8	98.3	100
75204	PBMC	4qA	10	16.2	0	7.1	12.5	23.2	39.3
75204	Saliva	4qA	10	14.1	5.4	8.9	13.4	14.3	26.8
75204	PBMC	DUX4 5'	10	50.8	6.8	35.6	58.5	64.4	91.5
75204	Saliva	DUX4 5'	12	59.3	5.1	31.4	74.6	78	88.1
75205	PBMC	4qA/10qA	10	80.6	66.7	77.8	80.6	85.2	96.3
75205	Saliva	4qA/10qA	9	84.1	66.7	75.8	85.2	92.5	92.6
75205	PBMC	DUX4 5'	10	71	40.7	57.6	73.7	86.4	88.1
75205	Saliva	DUX4 5'	10	63.9	32.2	55.9	61.9	78	86.4
RB19518	PBMC	4qA	6	10.1	5.4	7.1	9.8	14.3	14.3
RB19518	PBMC	DUX4 5'	6	15.5	5.1	6.8	19.5	20.3	22
